# Cone dystrophy and ectopic synaptogenesis in a *Cacna1f* loss of function model of congenital stationary night blindness (CSNB2A)

**DOI:** 10.1080/19336950.2017.1401688

**Published:** 2018-01-02

**Authors:** D. M. Waldner, N. C. Giraldo Sierra, S. Bonfield, L. Nguyen, I. S. Dimopoulos, Y. Sauvé, W. K. Stell, N. T. Bech-Hansen

**Affiliations:** a Department of Neuroscience, Cumming School of Medicine, University of Calgary, Calgary, Alberta, Canada; b Department of Medical Genetics, Cumming School of Medicine, University of Calgary, Calgary, Alberta, Canada; c Department of Ophthalmology and Visual Sciences, University of Alberta, Edmonton, Alberta, Canada; d Department of Physiology, University of Alberta, Edmonton, Alberta, Canada; e Department of Cell Biology and Anatomy and Department of Surgery, Hotchkiss Brain Institute, and Alberta Children's Hospital Research Institute, Cumming School of Medicine, University of Calgary, Calgary, Alberta, Canada; f Department of Medical Genetics, and Department of Surgery, Alberta Children's Hospital Research Institute, and Hotchkiss Brain Institute, Cumming School of Medicine, University of Calgary, Calgary, Alberta, Canada

**Keywords:** Cacna1f, Cav1.4, CSNB, retina, channelopathy, photoreceptor

## Abstract

Congenital stationary night blindness 2A (CSNB2A) is an X-linked retinal disorder, characterized by phenotypically variable signs and symptoms of impaired vision. CSNB2A is due to mutations in *CACNA1F*, which codes for the pore-forming α**_1F_** subunit of a L-type voltage-gated calcium channel, Ca_v_1.4. Mouse models of CSNB2A, used for characterizing the effects of various *Cacna1f* mutations, have revealed greater severity of defects than in human CSNB2A. Specifically, *Cacna1f*-knockout mice show an apparent lack of visual function, gradual retinal degeneration, and disruption of photoreceptor synaptic terminals. Several reports have also noted cone-specific disruptions, including axonal abnormalities, dystrophy, and cell death. We have explored further the involvement of cones in our ‘G305X’ mouse model of CSNB2A, which has a premature truncation, loss-of-function mutation in *Cacna1f*. We show that the expression of genes for several phototransduction-related cone markers is down-regulated, while that of several cellular stress- and damage-related markers is up-regulated; and that cone photoreceptor structure and photopic visual function – measured by immunohistochemistry, optokinetic response and electroretinography – deteriorate progressively with age. We also find that dystrophic cone axons establish synapse-like contacts with rod bipolar cell dendrites, which they normally do not contact in wild-type retinas – ectopically, among rod cell bodies in the outer nuclear layer. These data support a role for Ca_v_1.4 in cone synaptic development, cell viability, and synaptic transmission of cone-dependent visual signals. Although our novel finding of cone-to-rod-bipolar cell contacts in this mouse model of a retinal channelopathy may challenge current views of the role of Ca_v_1.4 in photopic vision, it also suggests a potential new target for restorative therapy.

## Introduction

Congenital stationary night blindness 2A (CSNB2A), an X-linked retinal disorder, is characterized by visual impairment including a phenotypically variable set of signs and symptoms. Important characteristics include defects of retinal neurotransmission in the outer plexiform layer, associated with impaired vision in dim (mesopic) illumination, reduced visual acuity, and increased risks of myopia, strabismus, nystagmus, photophobia and colour-vision defects [1,2]. CSNB2A is caused by mutations in *CACNA1F*, the gene that codes for the pore-forming α**_1F_** subunit of the L-type voltage-gated calcium channel, Ca_v_1.4 [3,4]. Ca_v_1.4 channels in the retina have been localized to rod and cone photoreceptor ribbon synapses, where they mediate graded glutamate release and the postsynaptic responses associated with visual signal transmission [5-7]. Despite the variability of dysfunction in Ca_v_1.4 channel properties [8], the postsynaptic ON- and OFF-bipolar cell components of the ERG (b- and d-wave, respectively) of CSNB2A patients are consistently reduced in amplitude [2,9,10].

Several murine models of CSNB2A have been used to characterize the effects of various *Cacna1f* mutations on retinal development, morphology and function [7,11-15]. Interestingly, loss-of-function mutant mice exhibit a more severe phenotype than their human counterparts, including a complete loss of visual-following behaviour (optokinetic response) and the post-photoreceptoral light-response (ERG b-wave) [14,16-19]. Immunohistochemical analyses of these mutant mouse retinas have shown that photoreceptor synaptic ribbons – structures highly specialized for continuous, high-output vesicular exocytosis – fail to mature in the absence of normal Ca_v_1.4 [7,20]. The well characterized morphological effects of this synaptic deficit include sprouting of horizontal and bipolar cell dendrites, extending past the outer plexiform layer (OPL) and into the outer nuclear layer (ONL) [14,15]; increased glial fibrillary acid protein (GFAP) reactivity in Müller cells, a known corollary of retinal stress [21,22]; and thinning of the outer nuclear layer (ONL), associated with photoreceptor death [14-16].

Recently, investigations of the Δ14-17 *Cacna1f*-KO model of CSNB2A revealed anomalies in the physiology of cone photoreceptors themselves. Cones in that model were reported to exhibit progressive degeneration after 6 months of age, and morphological abnormalities – including branching of cone axons – at an earlier age [16,20]. Preliminary observations in our *Cacna1f*-KO mouse model, G305X, have suggested that cones in this model may exhibit similar anomalies [15].

In light of the extensive variation in clinical features exhibited by individuals with different CSNB2A-causing mutations in *CACNA1F*, characterization of different murine ‘CSNB2A’ models is warranted. Therefore, to extend our characterization of the G305X model, to gain a deeper and more detailed understanding of the pathophysiology of cone photoreceptors in CSNB2A, and to assess the extent to which findings in the Δ14-17 loss-of-function model occur in our KO model, we investigated the retinas of G305X *Cacna1f*-KO mice with various techniques [14]. Using immunohistochemistry, TUNEL labeling, optokinetic response testing and electroretinography, we found that expression of several genes related to cone phototransduction was down-regulated, while genes related to cellular stress and deterioration were up-regulated, and that cone morphology and cone-mediated visual function progressively deteriorated, in line with previous evidence in the Δ14-17 *Cacna1f*-KO retina. Additionally, we show that sprouted PKCα-positive (ON-bipolar cell) dendrites form close appositions with dystrophic cone axons in the ONL, at sites where the cone axons contain presynaptic markers of ribbon synapses. No close contacts, synapse-like or otherwise, were ever observed between cones and PKCα-positive (putative rod-bipolar cell) dendrites in wild-type mouse retinas of the same age. In addition to demonstrating that the retinas of these two well-studied mouse models of CSNB2A are very similar in many respects, our new data provide novel insights into the plasticity of retinal circuitry in response to the developmental, neurohistological, and functional derangements in this model.

## Results

### Gene expression in the adult G305X retina

**Figure 1.**
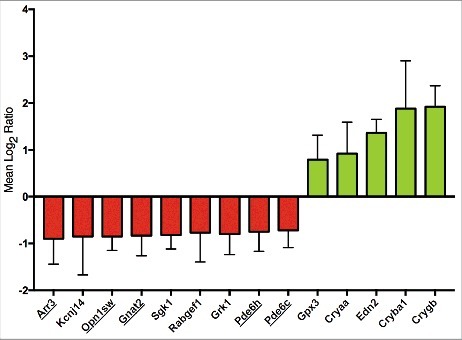
Mean Log_2_ ratio ±SD of differentially expressed genes identified via microarray analysis (Operon Microarray Mouse 17K 70-oligonucleotide Array) of adult G305X vs wildtype retinas. Cone-specific genes are underlined. All differentially regulated genes are described in Table 1.

Analysis of gene expression in wildtype and G305X adult retinas with the mouse Operon 17K Microarray chip revealed 14 annotated genes in which expression was either significantly down- or up-regulated, according to our criteria (Fig. 1). The identities and putative retinal functions of these genes are described in Table 1. Of the down-regulated genes, six of nine are involved in the cone phototransduction pathway, including cone arrestin (*Arr3*), S-Opsin (*Opn1sw*), guanine-nucleotide binding protein, alpha transducin (*Gnat2*), G-protein coupled receptor kinase 1 (*Grk1*), and the gamma and alpha-prime subunits of cone-specific phosphodiesterase (*Pde6h, Pde6c*). Genes that were significantly up-regulated included three members of the crystallin family (*Cryaa, Cryba1, Crygb*), endothelin-2 (*Edn2*), and glutathione peroxidase 3 (*Gpx3*), all of which are suggestive of biological stress and damage to the retina. The selective reduction of photoreceptor-specific genes led us to investigate early photoreceptor viability.

**Table 1.** Descriptions of differentially expressed genes in G305X versus wildtype mouse retinas at ∼3 months identified by microarray analysis.

Gene Name	Mean Log_2_**Ratio**	Standard Deviation	Fold Change	Putative Function in Retina	References
Cone Arrestin; *Arr3*	−0.9	0.54	−1.869	Regulation of cone opsins/phototransduction	[23]
Potassium Inwardly Rectifying Channel 2.4; *Kcnj14*	−0.85	0.82	−1.802	Regulation of membrane excitability/potential; neuronal activity	[29]
Short Wave Opsin; *Opn1sw*	−0.85	0.3	−1.802	GPCR; short wave/blue photon absorption	[33]
Guanine Nucleotide Binding Protein, Alpha Transducin 2; *Gnat2*	−0.83	0.43	−1.776	Transducin G protein, alpha subunit; couples cone opsin GPCRs to cGMP-phosphodiesterase (phototransduction)	[32]
Serum/Glucocorticoid Regulated Kinase 1; *Sgk1*	−0.82	0.66	−1.767	Regulation of ion channels/transporters	[28]
RAB Guanine Nucleotide Exchange Factor; *Rabgef1*	−0.77	0.62	−1.706	Endocytic membrane fusion/membrane trafficking	[30]
G Protein-Coupled Receptor Kinase 1; *Grk1*	−0.8	0.44	−1.742	Regulation of rhodopsin/cone opsins/phototransduction	[31]
Phosphodiesterase 6H; *Pde6h*	−0.75	0.42	−1.681	Phosphodiesterase, gamma subunit; Amplification of visual signal by cGMP production	[27]
Phosphodiesterase 6C; *Pde6c*	−0.73	1.11	−1.658	Phosphodiesterase, alpha-prime subunit; Amplification of visual signal by cGMP production	[27]
Gluthathione Peroxidase 3; *Gpx3*	0.79	0.52	1.729	Anti-oxidative enzyme; upregulated in retinal stress	[25]
Crystallin, alpha A; *Cryaa*	0.92	0.67	1.892	Chaperone-like properties; upregulated in cellular stress	[24]
Endothelin 2; *Edn2*	1.36	0.29	2.567	Vasoconstrictive signaling peptide; upregulated in photoreceptor stress	[26]

### Early apoptosis in the postnatal G305X retina

**Figure 2.**
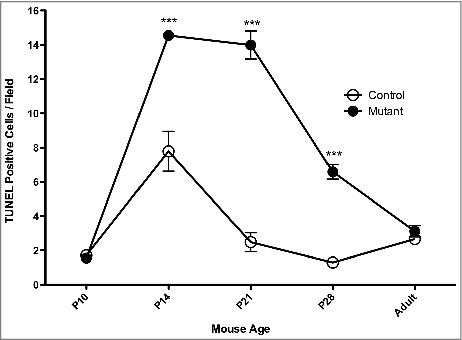
Quantitative analysis (Mean ± SD/Field) of TUNEL labelling in the outer nuclear layer of wild-type ‘Control’ (n = 3-5) and *Cacna1f-G305X* ‘Mutant’ (n = 4-5) mice at various postnatal ages (Adult = 3–5 months). **P < 0.05, ***P < 0.001.

A potential explanation for the down-regulation of cone-specific genes is loss of function, degeneration, or apoptosis of cone photoreceptors in the early post-natal G305X retina. To investigate this possibility, terminal deoxynucleotidyl transferase d-UTP nick end labeling (TUNEL) was performed, and the relative numbers of putatively apoptotic cells in the ONL were quantified, by counting labeled cells in a standard microscope field, in the developing retinas of wild-type and G305X mice of several ages (Fig. 2). At P14, P21 and P28 there were significantly more TUNEL-labeled nuclei in the ONL of mutant retinas than in those of wild-type controls (P14: *G305X =* 14.55 ± 0.22, wild-type = 7.79 ± 1.16; P21: G305X *=* 13.99 ± 0.82, wild-type = 2.48 ± 0.56; P28: G305X *=* 6.59 ± 0.43; wild-type = 1.30 ± 0.2 [nuclei/field ± SD]). Activation of apoptosis in ONL nuclei was confirmed at P14 by immunolabeling with an antibody to activated caspase-3, which labeled significantly more cells in this layer in G305X than in wildtype retinas (data not shown). As both cone and rod photoreceptor cell bodies are located in the ONL, and rods outnumber cones by more than 50:1, some of the apoptotic cells could be rod photoreceptors. Therefore, we sought to visualize abnormalities and signs of degeneration in cones – during this period of early apoptosis, and beyond, throughout the lifespan of these mice.

### Immunohistochemical analysis of G305X cone morphology

**Figure 3.**
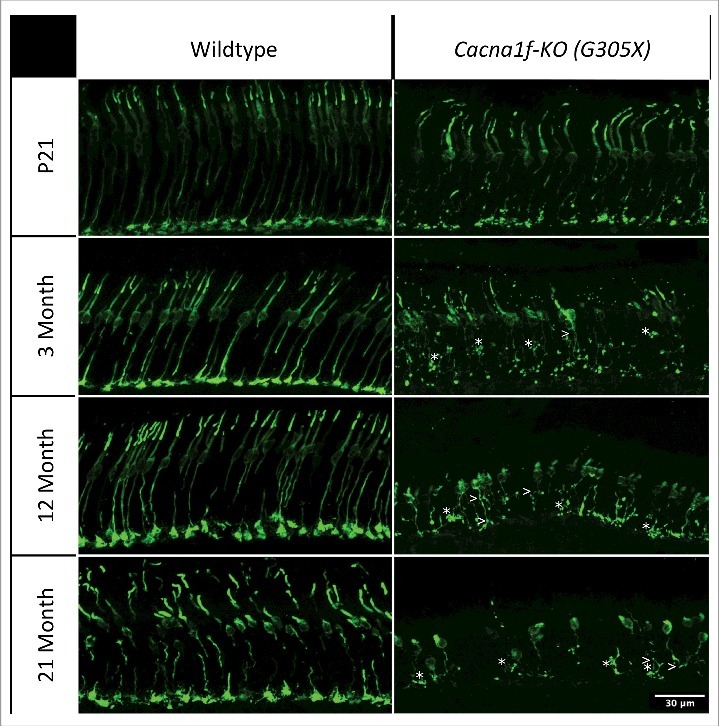
Cone arrestin (mCAR) immunolabeled age-matched wildtype and G305X mutant mouse retinas at P21, 3 months, 12 months and 21 months. G305X cones become increasingly dystrophic, and degenerate over time. Asterisks (*) and arrows (>) indicate axonal varicosities and branching in G305X retinas, respectively. Scale bar = 30 μm.

Previous studies, including our own, have shown that cones in *Cacna1f*-KO retinas become increasingly dystrophic and degenerate over time [15,16,20]. We sought to extend our characterization of the onset and progression of this degeneration by immunolabeling for an established cone-specific marker – cone arrestin (using antibody mCAR [36], which labels cones in their entirety) – at ∼21 days and 3, 12 and 21 months of age (Fig. 3). Cones in wildtype mice were uniform in structure, with synaptic pedicles aligned at a single level in the proximal OPL, at all ages – although they became mildly disordered at 21 months of age, as expected from published descriptions of age-related decline in structure and function of wild-type mouse cones.^37,38^ In contrast, the *Cacna1f*-knockout retinas were already dystrophic at P21, and they became increasingly so with advancing age. At P21, the G305X cone photoreceptors extended axons to the outer plexiform layer; their outer segments were mostly intact and regularly arranged, but their pedicles were structurally abnormal, with occasional fusiform varicosities along the otherwise uniformly thin cone axons (as observed in older G305X mice). At 3 months, outer segments were shortened or absent in the majority of G305X cones, and retraction, sprouting, and varicosities of cone axons in the outer nuclear layer were common. At 12 months, many cones lacked outer segments, and the degeneration of cone axons had progressed beyond the discrete, punctate pattern observed at 3 months. At the oldest age examined (21 months), numerous cones obviously had been lost, and the remaining cones were generally dystrophic – lacking outer segments, and having shrivelled and distorted axons without pedicles (Fig. 3).

**Figure 4.**
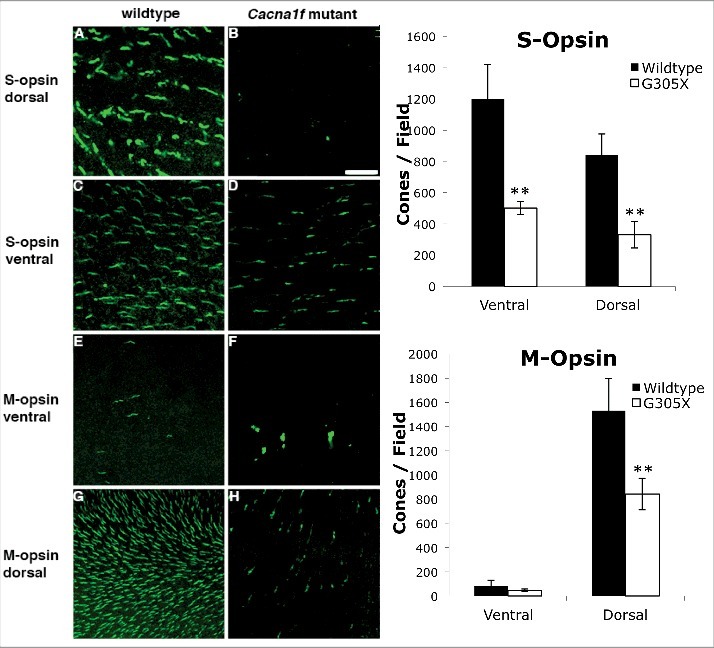
Left: S- (A-D) and M-Opsin (E-H) immunolabeled wholemounts of wildtype and G305X mutant mouse retinas visualized in ventral and dorsal regions. Differential distribution of cones in the murine retina is consistent with previous findings. [35] Right: G305X mice exhibit significantly fewer S- and M-opsin positive cones in three of four regions of retina; M-cones are rarely found in the ventral retina. Graphs represent mean cone counts (± SD), Field radius = 0.24 mm, **P < 0.01.

To reconfirm the loss of opsin-expressing outer segments, retinas from young adult mice (∼5 months) were whole-mounted and immunolabeled with antibodies to S- and M-opsins. Mouse cones contain short-wavelength- and middle-wavelength-sensitive opsins (S-opsin and M-opsin, respectively); most cones contain a mixture of the two [34], but for convenience we will refer to the cones labeled with anti-S-opsin as “S-cones”, and those labeled with anti-M-opsin as “M-cones”. Both cone types are distributed differentially across the normal mouse retina; S-cones are present throughout the entire retina, but at a higher density in the ventral retina, whereas M-cones are present mainly in the dorsal retina, and overall at a much lower density than S-cones [35]. Therefore, we counted outer segments expressing S- and M-opsins separately in dorsal and ventral retinal regions, in both wildtype and mutant retinas (Fig. 4). The population densities (numbers/field) of both S- and M-opsin positive outer segments were reduced throughout the mutant retina, most obviously in the areas of normally highest density. The density of outer segments in the mutant retina, compared to that in wild-type, for S-cones was 42% in the dorsal retina and 32% in the ventral retina, and for M-cones was 58% in the dorsal retina and 50% in the ventral retina. These results suggest that a global loss of cone outer segments and/or opsin content occurs in the G305X retina, between early postnatal and adult ages.

### Residual cones form synapse-like associations in the ONL of G305X mice

Previous studies have shown that bipolar and horizontal cell dendrites sprout, to extend past the outer plexiform layer into the outer nuclear layer, in *Cacna1f*-KO mice [14-16]. However, the features of these ectopic dendrites and their associations with other processes in the ONL have not yet been well characterized.

**Figure 5.**
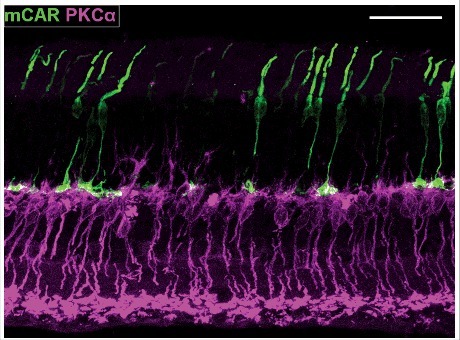
Cone arrestin (AF488)/PKCα (AF555) immunolabeled, ∼21 month carrier female (*Cacna1f^G305X^/Cacna1f^wt^)* retina; exhibiting putative wildtype–like and mutant–like regions in continuous field of the retina.

The *Cacna1f* gene is located on the X-chromosome (Xp11.23); therefore, heterozygous females are expected to exhibit a mosaic pattern of wildtype-like and mutant-like phenotypes across the retina, due to X-inactivation [17,39]. To confirm that the absence of α**_1F_** causes the phenotype of abnormal dendritic sprouting and cone photoreceptor degeneration in a spatially restricted manner in the G305X model (i.e., that the abnormality is specific to mutant cones), we co-immunolabeled retinas from ∼20 month-old heterozygous *Cacna1f^G305X/^*^wt^ carrier females – with antibodies to mCAR for visualization of cones, and antibodies to PKCα to visualize sprouting of ON- (putatively rod-) bipolar cell dendrites. Thus, as expected, in heterozygous retinas we observed radial columns having the mutant phenotype, with sprouting of bipolar cell dendrites and loss of cones – in contrast to the normal phenotype in contiguous columns of wildtype-like cells (Fig. 5). Notably, the vast majority of residual cones in these heterozygous retinas appeared rather normal, in contrast to the severely dystrophic cones, lacking outer segments, observed in homozygous G305X mutant retinas at about the same age (Fig. 3). That is: dystrophic cones – expected in the “mutant-like” columns – were rarely observed.

**Figure 6.**
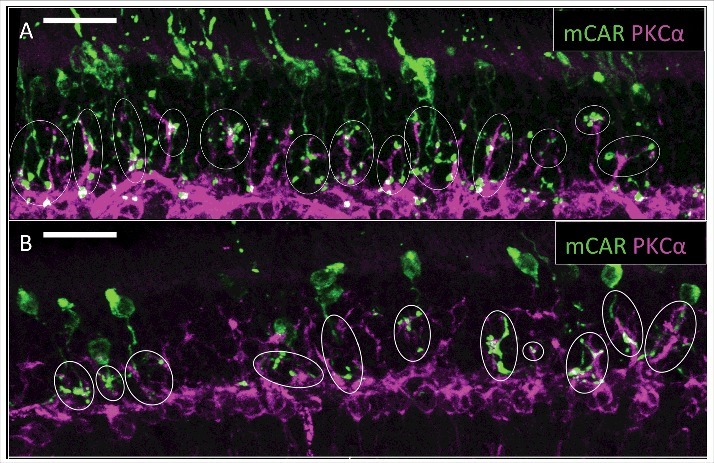
Cone arrestin (AF488)/PKCα (AF555) immunolabeled ∼3 month (A) and ∼21 month (B) G305X mutant retinas. White circles indicate ectopic cone and PKCα+ bipolar cell contacts. Scale bar = 20 μm.

**Figure 7.**
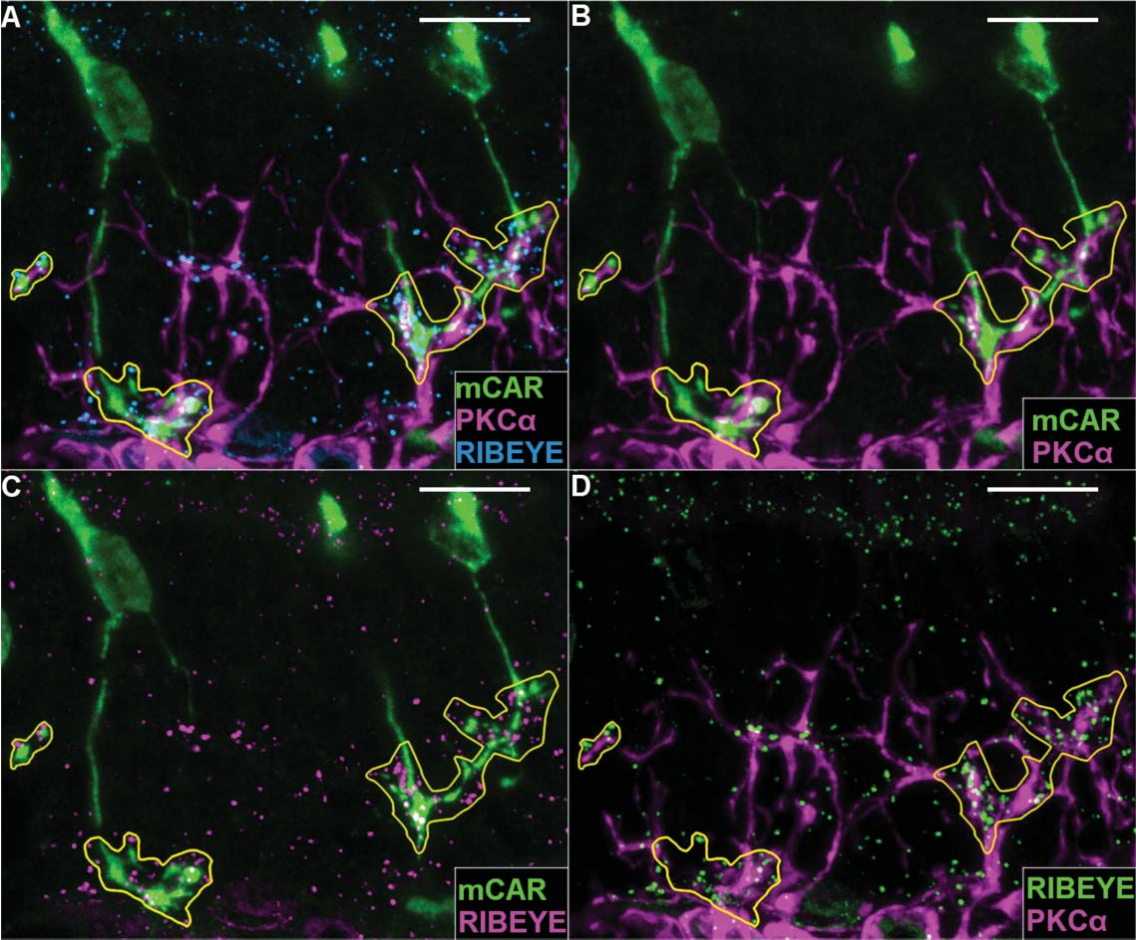
Cone arrestin (AF488)/PKCα (AF555)/RIBEYE (AF647) immunolabeled ∼21 month *Cacna1f*-KO retina. Yellow lines enclose regions of ectopic contact between mCAR positive cone processes and PKCα positive dendrites. Labels are shown collectively (A: mCAR – green, PKCα -magenta, RIBEYE – blue), and in pairs to increase visibility of co-localization (B: mCAR – green, PKCα – magenta; C: mCAR – green, RIBEYE – magenta; D: RIBEYE – green, PKCα – magenta). Scale bar = 10 μm.

**Figure 8.**
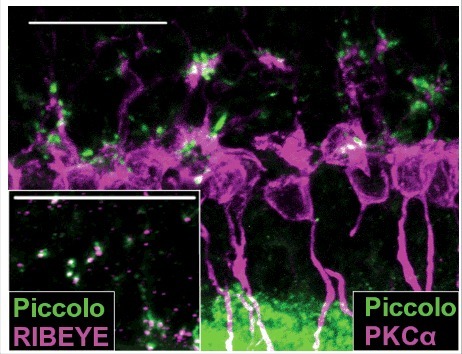
PKCα (AF555; magenta)/Piccolo (AF488; green) and Piccolo (AF488; green)/RIBEYE (AF555; magenta; inset) double-immunolabeled ∼21 month *Cacna1f*-KO retinas. Scale bar = 20 μm.

We performed similar analyses of wildtype and G305X retinas at different ages, to visualize relationships between the increasingly dystrophic cones and aberrant (PKCα-positive, presumably PKC-IR BC) bipolar cell dendrites. A previous study of the *nob2* mouse retina, in which wildtype-like α**_1F_** expression is ∼10% of normal [40] identified ectopic contacts between sprouted PKC-IR BC dendrites and rod photoreceptors; cones apparently do not become dystrophic in this model [41]. In our wildtype mice, cone pedicles and bipolar cell dendrites formed a monolayer in the proximal OPL at all ages, and cones were only minimally disordered at 21 months (data not shown). The mutant retinas, by contrast, were devoid of cone pedicles (see Fig. 3), but ectopic associations were observed between the aberrant PKC-IR BC dendrites and cone processes in the ONL. At 3 months, the atypical varicosities on cone axons were almost always associated with aberrant PKC-IR BC dendrites (12/12 clearly defined cones in Fig. 6A); and at 12 and 21 months, these associations had become increasingly close, with intertwining of the associated processes (Fig. 6B). In 21-month mutant retinas triple-labeled for mCAR, PKCα and the ribbon-associated presynaptic marker RIBEYE, regions of interaction of dystrophic cone processes and sprouted rod bipolar cell dendrites exhibited increased packing-density of RIBEYE-positive puncta, suggesting these contacts might be functionally synaptic (Fig. 7). Further labeling experiments revealed that piccolo (another ribbon-associated presynaptic marker) also is present at these points of contact, as previously reported in the Δ14-17 *Cacna1f*-KO model^7^ (Fig. 8). Given these suggestions of synaptic contact between residual (though dystrophic) cones and rod-bipolar cells in our G305X mice, we undertook further studies of visual function to test for synaptic transmission at these contacts.

### Characterization of cone-dependent visual function (Optokinetic Response)

**Figure 9.**
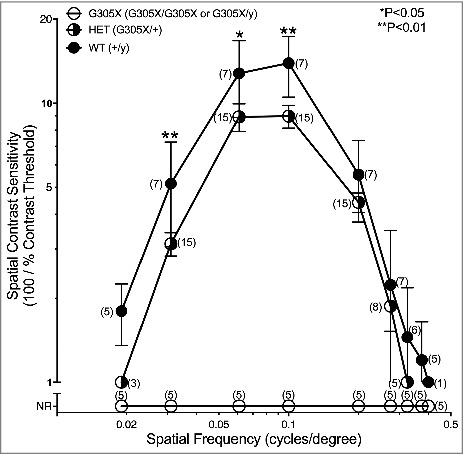
Optokinetic response analysis. Mean photopic spatial contrast sensitivities (± SD) of wildtype, G305X heterozygous (*Cacna1f^G305X^/Cacna1f^wt^)* carrier females and G305X mutant mice. This analysis includes data from Lodha et al. 2010^18^. Bracketed numbers indicate n-value of each data point. *P < 0.05, **P < 0.01.

As described previously, the photopic *spatial contrast sensitivity function (CSF)* of C57Bl/6J mice (contrast sensitivity [CS] as a function of spatial frequency [SF]) is in the form of an inverted “U”, with CS reaching a maximum at intermediate SFs and falling off rapidly at higher and lower SFs [18,42]. At drift velocity (V) = 12 d/s, CS was maximal at SF = 0.061-0.100 cycles/degree (c/d) and fell rapidly to CS = 1 above SF = 0.400 c/d and below SF = 0.013; no optokinetic responses were evoked outside this range of optokinetically “visible” SFs, even at maximum luminance and contrast. In line with previous observations, mutant mice (*Cacna1f^G3O5X^/Y* and *Cacna1f^G3O5X^/Cacna1f^G305X^*) were optokinetically blind, failing to follow gratings at any V and SF [18]. Heterozygous (carrier) *Cacna1f^G305X^/Cacna1f^wt^* females exhibited only moderately reduced optokinetic responses (Fig. 9). The shape of the CSF in heterozygous females was similar to that in wildtype C57BL/6J mice, except for an overall reduction in contrast sensitivity to 56–83% of the wildtype values (average 72% over the seven SFs tested from 0.019-0.325 c/d). As a result, CS at optimal SF (0.100 c/d) averaged 8.87 ± 3.86 in heterozygous females, compared to 12.2 ± 4.69 in wildtype mice (P < 0.01); furthermore, the SF range was reduced somewhat: to 0.19-0.325 c/d in heterozygotes, compared to 0.013-0.400 c/d in wildtypes. Regression analysis revealed no significant reduction in CS with ageing, in wildtype or heterozygous mice (data not shown). Thus the results of photopic OKR testing suggest a lack of functional neurotransmission at the ectopic cone-to-PKC-IR BC contacts, under photopic conditions. To further substantiate these findings, electroretinographic analysis was performed.

### Electroretinography

**Figure 10.**
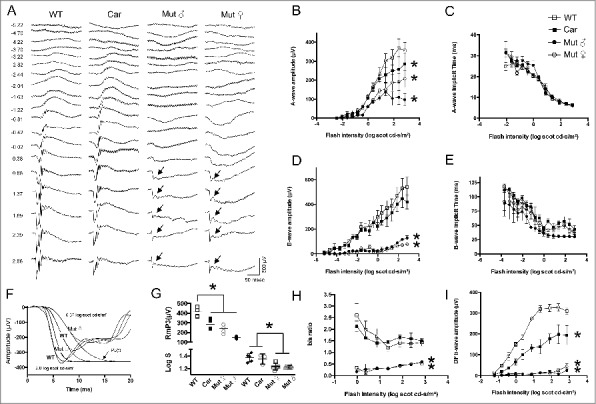
Dark-adapted ERG. A) Representative traces elicited from single flashes of incremental intensity in the four groups studied. Arrows indicate partial b-waves. Units on left represent flash intensity (log cds/m^2^). B-E) Intensity response results (n = 6 per group) for a-wave amplitude (B) and implicit time (C) and for b-wave amplitude (D) and implicit time (E). F-G) Modeling of a-wave leading edge, with representative traces of actual responses (continuous lines) versus modeled traces (dotted lines) in panel F; values for gain parameter (Log S) and for maximum rod photoresponse amplitude (RmP3) are provided in panel G. Panel H shows amplitude ratios of post-synaptic (b-wave) over pre-synaptic (a-wave) retina functional markers as a function of flash intensity. Finally, panel I provides the amplitude of dark-adapted rod b-waves (obtained by subtracting the intensity-matched single flash mixed b-wave by the double-flash isolated cone response) as a function of the second flash intensity. WT = wildtype; Car = G305X mutant heterozygous (carrier) female; Mut ♂ = G305X mutant males and Mut ♀ = G305X mutant females. Individual points on graphs represent averages ± SD; asterisks indicate statistical significance at p < 0.05.

We further investigated retinal function using detailed electroretinographic testing of wildtype, heterozygous (carrier) and *G305X* mutant mice. Under dark adaptation (Fig. 10), partial b-waves could be recorded in G305X mutant female and male mice only at high flash intensities (see arrows) corresponding to the range of mixed rod and cone responses. Carriers (heterozygous females) showed a specific reduction in a-wave amplitude, compared to WT. Mutant mice had reduced a-wave amplitudes compared to WT, and b-waves exceeding the 20 µV criterion amplitude only at the five highest flash intensities. There were no differences in implicit times between groups. The reduction of the b-wave:a-wave ratio [b-wave amplitude (post-synaptic function) over a-wave amplitude (pre-synaptic function)], in both mutant males and females compared to WT and carriers, confirmed the impairment of synaptic transmission in the mutant retinas. Double-flash isolation of pure rod-driven b-waves, over the tested range of 19 flash intensities, confirmed that rod-to-PKC-IR BC transmission is not only severely depressed in mutant mice, but also defective in carrier females. Mathematical modeling of the leading edge of the a-wave yielded reduced RmP3 (maximum rod photoreceptor amplitude) in mutant and carrier mice compared to WT mice, pointing to deterioration of rod photoreceptor function. The other parameter obtained from this modeling, log S (photoreceptor gain), was reduced in both male and female mutants compared to that seen in WT and carriers, indicating major loss of photoreceptor sensitivity in the G305X mutant retina.

**Figure 11.**
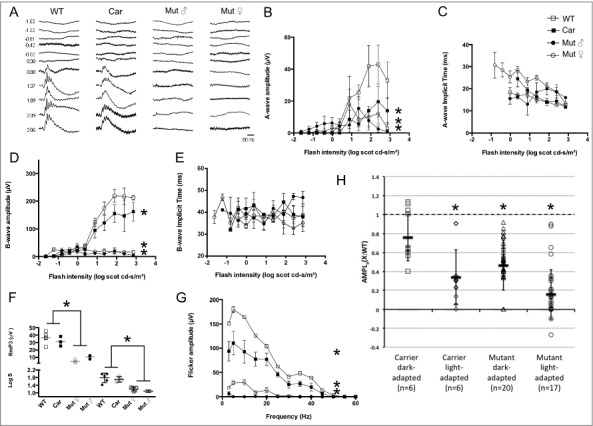
Light-adapted ERG. A) Representative traces elicited from single flashes of incremental intensity in the four genotypic groups studied. Mutant males and females have extinguished photopic ERGs. Units on left represent flash intensity (log cds/m^2^). B-E) Intensity response results (n = 6 per group) for a-wave amplitude (B) and implicit time (C) and for b-wave amplitude (D) and implicit time (E). F) Values for gain parameter (Log S) and for maximum cone photoresponse amplitude (RmP3) obtained from modeling the leading edge of the photopic a-wave. G) Flicker amplitude as a function of flash frequency. H) Maximal photoresponse amplitude normalized against that of WT as obtained under dark- and light-adapting. WT = wildtype; Car = G305X mutant heterozygous (carrier) female; Mut ♂ = G305X mutant males and Mut ♀ = G305X mutant females. Individual points on graphs represent averages ± SD; asterisks indicate statistical significance at p < 0.05.

Under light adaptation (Fig. 11), cone-driven photopic a-wave amplitudes reached the 20 µV criterion threshold only in WT. In addition to sub-threshold a-wave amplitudes, the carrier group also showed reduced cone-driven b-wave amplitudes compared to WT. The only difference in implicit times (calculated for all responses including sub-threshold ones) was that for a-waves in the G305X mutant females. Flicker amplitudes, as a function of flash frequency, did not exceed the 20 µV criterion threshold in either male or female mutants, and the flicker responses at higher frequencies (30-45Hz) were diminished in carriers compared to WT.

Finally, plotting of maximal photoresponse amplitude (RmP3), normalized against that of WT (as obtained under dark- versus light-adapting background), showed that photopic (cone-driven) responses were more affected than mesopic (mixed rod-cone) responses.

## Discussion

In these studies, we have found severe functional and morphological changes in cone photoreceptors in the retina of the ‘G305X’ mouse model of CSNB2A.

Genetic microanalysis yielded several important insights into the effects of *Cacna1f* knockout in G305X mutant retinas – most significantly the selective down-regulation of several cone-specific genes (Fig. 1). Down-regulation of genes in the cone phototransduction pathway has been reported in different mouse models of retinal degeneration, including *Cnga3^−^^/^^−^/Nrl^−^^/−^* (cone-enriched) mice, in which photoreceptors are incapable of light-induced hyperpolarization [43,44]. Mouse *Cnga3^−^^/^^−^* models exhibit phenotypic similarity to *Cacna1f^−^^/^^−^* (G305X) mice, including sprouting and extension of horizontal and bipolar cell dendrites into the ONL [45]. The down-regulation of cone gene expression in our mice may be due in part to early photoreceptor apoptosis, as revealed in our TUNEL labeling (Fig. 2); a similar phenomenon was also observed in the Δ14-17 *Cacna1f*-KO model [16]. The absence of a parallel decrease in rod-specific gene expression may be due to the loss of only a small fraction of the large rod population, in contrast to the loss of a large fraction of the small cone population. Immunohistochemical analyses, however, suggest that other factors beyond cone death contribute to down-regulation of genes in the G305X model. Cone arrestin immunoreactivity, for example, is known to be severely down-regulated in residual cones in *Cacna1f*-knockout models, prompting the use of antibodies to less common cone-specific proteins such as glycogen phosphorylase [20,46]. Although not definitively shown in the present study, a potential mechanism for cone-selective gene down-regulation and/or apoptosis involves the unfolded protein response – previously implicated in retinal degeneration [47]. Cone pedicles in mice contain ∼10 synaptic ribbons, whereas rod spherules contain only one [48]. Thus, as Ca_v_1.4 is associated with the arciform density, which in turn is related 1:1 to a synaptic ribbon [7], cones may normally transcribe *Cacna1f* ∼10x more rapidly in cones than in rods, making cones more susceptible to *Cacna1f^G305X^-*mediated unfolded protein response or other effects of α**_1F_**-protein truncation. The up-regulated genes identified here, including those for three crystallins, glutathione peroxidase 3 and endothelin 2, have all been implicated in retinal stress responses, serving diverse functions in different stress models [24-26,49]. Further research into changes of gene expression, as well as the functional roles of these genes and their up-regulation, may help us to understand better the retinal pathophysiology in *Cacna1f* mutants.

TUNEL labeling in early postnatal G305X retinas showed a significant increase in prevalence of apoptotic cells, beginning at P14 and sustained through P21/P28, with no significant increase observed in adulthood compared to wildtype controls. These data align with observations in P28 and adult retinas of Δ14-17 *Cacna1f*-KO mice [16], but also suggest that increased early apoptosis due to *Cacna1f*-KO does not begin until the period during which ribbon synapses normally develop. Increased apoptosis is a common feature of neurons that fail to signal early after synaptogenesis; elsewhere in the CNS, for example, it has been observed in the thalamus, hippocampus, and fimbria of *Munc 18-1* knockout mice, which do not secrete neurotransmitter despite having formed structurally normal-appearing synapses [50]. It is not clear why relative rates of photoreceptor cell death decrease beyond P21 into early adulthood.

Visualization of cones in retinal cross-sections at various ages showed progressive degeneration and dystrophy, as well as distinct abnormalities (Fig. 3). The pronounced irregularities in cone axons, which we observed with mCAR labeling, have also been described in the *Δ14-17 Cacna1f*-KO and the *rd1* mouse models. It was suggested that these events might be precursors of cone death in the *rd1* mouse [51]. These abnormalities precede the reduction in spatial cone densities seen in cross sections at later ages, which align with previous studies of the Δ14-17 *Cacna1f*-KO model which observed no significant loss of cones until >6 months of age [16,20]. The gradual, global loss of outer segments (visualized by M- and S-opsin labeling at ∼5 months in our study – Fig. 4) suggests that this loss may also precede cell death in cones of the G305X retina.

The relative rarity of axonal abnormalities, or any other signs of cone dystrophy, in aged heterozygous G305X mice – in which all remnant cones appear “healthy” – is particularly intriguing. Mutant-like columns in ∼21-month heterozygous retinas have fewer cones, but the residual cones do not exhibit the dystrophic characteristics of cones in mutant G305X retinas at the same age. Michalakis et al. suggest that second-order neurons in mutant-like columns form synapses with bordering photoreceptors in wildtype-like columns. As a result, cones in the mutant-like columns may receive fewer ectopic interactions and thus degenerate more rapidly, leading to the absence of dystrophic cones in mutant-like regions [17]. This hypothesis supposes that ectopic interactions between second-order neurons and photoreceptors increase cone viability in the G305X mutant retina. Alternatively, the presence of wildtype-like columns may contribute to the preservation of cone morphology in adjacent mutant-like columns.

The observation of ectopic, synapse-like contacts between PKC-IR BC dendrites and dystrophic cones in the ONL of aged G305X retina is a novel finding in a mouse model of CSNB2A. Ectopic synaptogenesis was previously described in several retinal degeneration models, however, involving various connections: between cones and PKC-IR BCs in the OPL of *rd1* and *Rho^−^^/^^−^* mice (both of which have rod-specific dysfunction); between rods and cone-BCs in *CNGA3^−^^/−^* mice (with cone-specific dysfunction); and between cones and horizontal cells in the ONL of *Δ14-17 Cacna1f*-KO mice [20,45,52,53]. However, although similar contacts have been described in retinas of WT mice at advanced ages, the current observation of ectopic synapse-like contacts between cones and PKC-IR BCs (Figs. 6, 7 and 8) is the first evidence, to our knowledge, of such contacts in the ONL of a mouse retinal degeneration model [38]. The absence of such apparently ‘synaptic’ contacts in younger G305X mice aligns with observations in *CNGA3^−^^/^^−^;Rho^−^^/^^−^* mice, in which no ectopic synaptogenesis was observed by P46 – though it remains to be seen whether they develop with further aging in that model [45]. Ectopic synapses in the ONL, between rods and both horizontal and rod-bipolar cells, have been observed in the *Cacna1f-nob2* mouse model, in which ∼10% of the normal amount of wildtype-like α**_1F_** protein is produced [13,40]. Electron-microscopical studies of *nob2* retinas suggest that these contacts are exclusively between rods and second-order neurons, and that cone terminals are not so severely affected as in other, pure *Cacna1f*-KO models [41]. Despite the presence of synaptic markers at the ectopic contacts in G305X retinas, functional studies suggest that these synapses either are purely structural, or contribute only to a sub-threshold visual response for ERG and OKR analysis. One might also consider the possibilities, perhaps remote, e.g.: that PKC-IR BCs in mutant mice (a) become desensitized under photopic conditions, (b) are in some other way made unresponsive to cone input by the G305X mutation, or (c) do not feed into the DS-RGC circuit that drives the OKR. Further investigation, including ultrastructural characterization of these contacts and functional studies of the ON-DS-RGCs that drive the OKR, will help to elucidate the underlying mechanisms.

The decrease in ERG b-/a-wave amplitude ratios, under dark- and light-adaptation, is evidence of defects in post-photoreceptoral activation of both rod and cone ON-bipolar cells in G305X mutant males and females (Figs. 10 and 11). The results of double-flash stimulation (to isolate purely cone-driven b-waves) further support the interpretation that rod to PKC-IR BC transmission is severely depressed – not only in the mutants, but also in the female carriers. In fact, the small partial b-waves observed may not be reflective of limited residual BC activation, but instead may be generated by photoreceptor activity as reported in Mansergh et al. [14] Our ERG findings also indicate that the pre-synaptic elements (rod and cone photoreceptors, respectively) not only show features of synaptopathy, but also undergo degeneration (cell death), in all groups – as previously reported for CORDX3-like phenotypes in humans, caused by deleterious mutations in *CACNA1F* – by our group [54] and more recently by others [55]. The reduction in RmP3 values (maximum rod photoreceptor amplitude) compared to those in WT, in all three models (mutant female, mutant male, and carriers), implies that rods undergo degeneration. In contrast to these findings in our G305X mice, dark-adapted RmP3s were found to remain normal in the *nob* mouse – suggesting that rods do not degenerate in the *nob* model, in which the defect (in the nyctalopin gene, *Nyx*) is *post*synaptic in the photoreceptor ribbon synapses [13,56]. We also found a reduction in light-adapted a-wave amplitudes (not exceeding the 20 µV criterion threshold), implying that cones also undergo degeneration in the G305X mutant mice and carrier females studied here (Fig. 11). Furthermore, the reduction in log S (photoreceptor gain parameter) indicates that the majority of surviving rods exhibit decreased amplification and decreased efficiency of initial phototransduction stages, in both male and female G305X mutant mice, compared to WT and carriers. Similarly, prolonged light-adapted a-wave implicit times also indicate a biochemical defect in the residual cones of G305X mutants. Modeling of leading-edge a-wave kinetics (both cone- and rod-driven), in human CSNB2A (due to *CACNA1F* mutations, but not necessarily comparable to the early truncation G305X mutation), did not reveal changes in photoreceptor sensitivity [57]. Finally, plotting of maximal photoresponse amplitudes (RmP3) in our G305X mice, normalized against those of WT (as obtained under dark- versus light-adapting background) mice, shows that cone-driven responses are more affected than mixed rod-cone responses. These observations support the conclusion that cone death is more pronounced than rod death, in both male and female G305X mutant mice, thus confirming our histological and immunohistochemical observations.

Female carriers of loss-of-function mutations in *Cacna1f* (mouse)/*CACNA1F* (human) exhibit some mild symptoms [17,58,59]. Our immunohistochemical observations, combined with the detailed ERG and OKR functional analyses, support the existing literature – suggesting that mosaic X-inactivation in the heterozygous retina results in only relatively mild impairment of visual function (Fig. 5) [17,39]. This may be grounds for optimism – that gene therapies may be highly effective in restoring substantial levels of visual function, even without achieving full transduction of all affected cells in CSNB2A-afflicted retinas.

## Materials and methods

### Experimental animals

The generation of G305X *Cacna1f*-KO mice was described previously. These mice exhibit premature truncation of the α_1F_ protein due to a glycine to stop codon mutation introduced via a neocassette insertion into exon 7 corresponding to codon 305 of *Cacna1f* [14]. Both male (*Cacna1f^G305X^*/Y) and female (*Cacna1f^G305X^/Cacna1f^G305X^)* mutant and wild-type mice were used; carrier females were heterozygous for the G305X mutation (*Cacna1f^G305X^*/*Cacna1f^wt^*). Mice were maintained in the Mouse Double Barrier Unit (MDBU) of the Clara Christie Centre for Mouse Genomics (CCCMG) under a 12:12 hour light:dark cycle. All of our mouse experiments were approved by the University of Calgary Animal Care Committee and complied with the ARVO Resolution on the Care and Use of Animals for Research.

### Gene expression microarray analysis

Total RNA was isolated from retinas of adult (3-5 months) C57BL/6J wildtype and *G305X* mice age-matched littermate mice, using Trizol Reagent (Invitrogen, https://www.thermofisher.com/order/catalog/product/15596026), and treated with the MessageClean kit (GenHunter, http://www.genhunter.com/products/messageclean-kit-rna-cleaning.html) to remove contaminating DNA. RNA from 10 adult animals of each genotype was combined to reduce effects of individual variation. cDNA was then synthesized, coupled to Cy3 or Cy5 dye, and purified, using the Superscript Indirect cDNA Labeling System (Invitrogen, https://www.thermofisher.com/order/catalog/product/L101402). Cy3- and Cy5-labeled cDNA samples were appropriately diluted in DIG Easy Hyb solution (Roche Life Sciences, http://www.sigmaaldrich.com/catalog/product/roche/11603558001) containing 5% (v/v) each of yeast RNA and salmon sperm DNA as blocking agents. The hybridization solutions were then applied to Operon Microarray Mouse 17K 70-oligonucleotide array chips (printed at the Southern Alberta Microarray Facility, University of Calgary), and incubated for 18 hours at 37°C. Four experiments were conducted using total RNA isolated and pooled from ten mutant and ten wildtype control retinae for each experiment, including dye-swap hybridizations to reduce variability from dye incorporation and fluorescence intensity. Scanning of the oligonucleotide microarray hybridized slides was performed with a Virtek Chip Reader (Virtek Biotech Canada Inc.), and fluorescence signals were quantified using the QuantArray version 3.0 microarray analysis software (Packard Bioscience, Billerica MA). Normalization and data analysis were performed with the GeneTraffic software (Iobion Informatics, La Jolla, CA), excluding genes with fewer than 10 valid spots and/or a coefficient of variance greater than one. Up- and down-regulated genes were defined as having a minimum mean log_2_ ratio of ±0.67 compared to control tissues.

### Immunohistochemistry and TUNEL Labeling

Wildtype and G305X mice of different ages were euthanized with pentobarbital sodium (Euthanyl, Bimeda-MTC), 0.1mL/225g body weight; eyes were enucleated, cornea and lens were removed, and the posterior eyecup was fixed in 4% paraformaldehyde–3% sucrose in 0.1 M phosphate buffer, pH 7.4 (137mM NaCl, 2.7mM KCl, 10 mM Na_2_HPO_4_, 2 mM KH_2_PO_4_) for ∼30 min @20°C. For retinal sections, eyecups were washed in phosphate-buffered saline (PBS), cryoprotected overnight in 30% (w/v) sucrose, mounted in O.C.T. Compound (Sakura Finetek Inc., http://www.sakura.eu/Our-products/item/11/Cryotomy/48/Tissue-Tek-OCT-Compound-and-Cryomolds), and cryosectioned at 12 µm. Sections were thaw-mounted onto slides, air-dried, and stored at −20°C until ready for antibody or TUNEL labelling. For whole-mount preparations, retinas were removed from the eyecup after the initial fixation and fixed for an additional 30 minutes, before overnight incubation in a 30% (w/v) sucrose solution.

Slides bearing retinal sections were initially incubated in blocking solution (0.5% normal goat serum, 0.5% Triton X-100 in PBS) for 30 minutes, followed by overnight incubation in diluted primary antibody (See Table 2) in the same solution at room temperature, followed by a 2-hour incubation in secondary antibody the following day. Whole-mounted retinas were pre-incubated in blocking solution for 3 hours, then incubated in primary antibody for 3 days, and secondary antibody for one day at 4°C. The secondary antibodies used were AlexaFluor 555-, 647- or 488-conjugated donkey anti-mouse, anti-goat or anti-rabbit IgG (Jackson ImmunoResearch), diluted 1:500. TUNEL labeling of retinal sections was performed by incubating slides in TUNEL incubation solution (15 U 3′ terminal deoxynucleotidyl terminal transferase (Invitrogen, https://www.thermofisher.com/order/catalog/product/EP0161), 0.25 nmol Cy3-conjugated dCTP (GE Biosciences, http://www.gelifesciences.com/webapp/wcs/stores/servlet/productById/en/GELifeSciences/25005441), 500 mM potassium cacodylate (pH 7.2), 10 mM CoCl_2_ and 1 mM DTT in PBS) for one hour at 37°C. Cross-sections were viewed and images were captured on an Olympus FV1000 laser-scanning confocal microscope with a 60x objective (1.42 oil, PlanApoN) in the Snyder Institute (University of Calgary) Live Cell Imaging Facility, as Z-stacks at Nyquist resolution. In double- and triple-labeling experiments – using two or three primary antibodies, visualized by secondary antibodies labeled with different ALEXA-Fluor dyes – control experiments confirmed that the multiple labels were distinguished reliably by the filters or laser settings used. Digital image processing to improve sharpness and contrast for clarity of presentation and z-stack projections (Fig. 4, Fig. 5, Fig. 6, and Fig. 7) were performed using Image J 1.47. Wholemount images were captured via epifluorescence microscopy (Zeiss, 25x/0.8 NA Plan-Neofluar). Statistical significance of differences in whole-mount M- and S-opsin-positive cone counts and TUNEL-positive cells, between wildtype and G305X mutant retinas, was calculated using two-tailed Student's unpaired t-tests in Prism™ (GraphPad Software Inc., San Diego, CA, USA).

**Table 2.** Primary antibodies used in this study.

Antibody	Host Species	Form	Dilution	Source	Antigen	References
**Cone Arrestin**	Rabbit	Affinity Purified Polyclonal Antisera	1:1000	C. Craft, Univ. Southern California	bovine CRX oligopeptide (279-292)	[36]
**M-Cone Opsin**	Rabbit	Affinity Purified Polyclonal Antisera	1:2000	C. Craft, Univ. Southern California	mouse M-opsin oligopeptide (3–16)	[61]
**S-Cone Opsin**	Rabbit	Affinity Purified Polyclonal Antisera	1:2000	C. Craft, Univ. Southern California	mouse S-opsin oligopeptide (1–11)	[61]
**PKCα**	Mouse	Purified Mouse Monoclonal IgG2a	1:1000	Cedarlane Labs, Burlington, ON	purified bovine brain PKCα (Clone MC5)	[63]
**PKCα**	Goat	Purified Goat Polyclonal IgG	1:1000	R&D Systems, Minneapolis, MN	Recombinant human PKCα (604-672)	[63]
**RIBEYE**	Mouse	Purified Mouse Monoclonal IgG	1:1000	BD Biosciences, Mississauga, ON	mouse RIBEYE oligopeptide (361-445) (Clone 16/CtBP2)	[62]
**Piccolo**	Rabbit	Affinity Purified Polyclonal Antisera	1:1000	Abcam, Eugene, OR	rat Piccolo oligopeptide (within residues 600 – 700)	[60]

### Behavioural analysis of visual function

Measurement of the photopic optokinetic contrast sensitivity of wildtype, heterozygous and G305X mice was performed using the OptoMotry™ system, as previously described [18,42,64]. Horizontally drifting vertical sine-wave gratings, drifting to either left or right, were presented at various spatial frequencies (SF: in steps from 0.003 cycles/degree[c/d] to 2.0 c/d) at constant drift velocity (V: 12 degrees/second – previously determined to be optimal for C57Bl/6 mice [42]. To determine the spatial contrast threshold, spatial luminance contrast (Michelson contrast [M_c_ = (L_max_ – L_min_) / (L_max_ + L_min_)]) was varied stepwise from above and below, by a modified staircase procedure (‘incorrect’ responses led to re-testing at the same contrast), between 0 and 100%; L_max_ = 104 cd/m^2^, L_min_ = 4 cd/m^2^, and L_mean_ = 54 cd/m^2^ (measured by Minolta LS-110 Luminance Meter in spot mode). Thus the luminance was always within the photopic range (∼1,000-10,000x, or 3–4 log units above mesopic range), which in C57BL/6J mice begins at about 0.01 cd/m^2^ (Umino et al., 2008). Percent contrast at threshold (Thr) was converted to contrast sensitivity (CS; dimensionless), by calculating CS = 100/Thr. Differences in contrast sensitivity between two groups, at a given spatial frequency, were assessed by a two-tailed Student's *t*-test in Prism™ (GraphPad Software Inc., San Diego, CA, USA.)

### Electroretinographic (ERG) analysis of retinal function

ERGs were recorded as previously described [65] from mutant G305X female mice (homozygous, *Cacna1f^G305X^/Cacna1f^G305X^;* n = 20), mutant G305X male mice (hemizygous, *Cacna1f^G305X^/Y;* n = 6), female carriers (heterozygous, *Cacna1f^G305X^/Cacna1f^wt^;* n = 6) and age-matched wildtype mice (*Cacna1f^wt^/Cacna1f^wt^* or *Cacna1f^wt^/Y;* n = 6). Following overnight dark-adaptation, mice were prepared for bilateral recordings under dim red light. While under anaesthesia (Xylazine 10 mg/kg i.p; ketamine 150 mg/kg i.p.), body temperature was maintained at 38°C with a homeothermic blanket, and both pupils were dilated with topical 1% Tropicamide. The corneas were kept hydrated by applying drops of an eye lubricant (Optixcare, Aventix, http://www.optixcare.ca/product-eyelube.htm), which also contains electrolytes, thus providing electrical contact with the recording electrode (gold wire loop). Reference electrodes were a pair of 25-gauge platinum needles inserted subdermally behind each eye. Amplification (0.03-1000 Hz bandpass), stimulus presentation, and data acquisition were provided by the Espion E^2^ ERG system (Diagnosys LLC, Lowell, MA). Under dark-adaptation, tests began with single flashes (6500K, 10 µs duration) presented at nineteen increasing luminance steps ranging from –5.22 to +2.86 log cd/m^2^. The inter-stimuli-interval (ISI) was progressively increased from 5 sec (at the lowest intensity) to 2 minutes (at the highest intensity), to minimize rod photopigment bleaching between the 3 to 6 responses required to obtain reliable response averages. The amplitude of the b-wave was measured from the a-wave negative peak to the b-wave positive apex, rather than to the peak of oscillations, which can exceed the peak of the b-wave.

Rod and cone responses were isolated with a double-flash protocol, as previously described [66]. In brief, a probe flash was presented 800 msec after a conditioning flash (1.4 log cd/m^2^); this transiently saturated rods so that only cones were responsive to the probe flash. Rod-driven b-waves were derived by subtracting the cone-driven response from an intensity-matched single-flash mixed response. By varying the intensity of the probe flash it was possible to obtain isolated cone intensity-response functions. Averages of up to 3 traces were obtained for each stimulus condition; intervals between double flash presentations were set at 2 minutes to optimize recovery of rod responsiveness. Following 10 min of light-adaptation (30 cd/m^2^ background), cone-driven photopic intensity-response functions were obtained, using single flashes (6500K, 10 µs duration) presented at eleven steps of increasing luminance, varying from −1.63 to +2.86 log cd/m^2^. Trace averages were derived from 6 responses elicited at 5 sec intervals. Values on graphs represent averages ± standard deviations.

Finally, to quantify parameters of phototransduction activation, a-waves elicited by the four highest stimulus intensities (1.37, 1.89, 2.39, and 2.86 log cd/m^2^) were fitted with the Hood and Birch equation [67], of the Lamb and Pugh phototransduction activation model [68] which describes the response (R) as a function of flash intensity (I), and time (t):

$$R\left(I,t\right)=\left\{1-e^{\left[-I\cdot S\cdot {\left(t-td\right)}^{2}\right]}\cdot Rmp3\right\}$$

Two main parameters are derived from this model: the amplitude of the saturated rod response (Rmp3; μV) and the sensitivity parameter (S; m2 cd-1 s-3). The brief delay before the onset of the a-wave is taken into account as td. Best fitting values of Rmp3 and S were estimated using a Chi-Square minimization curve-fitting method in Igor Pro (Wavemetrics, Inc., Lake Oswego, OR). Fitting was restricted to the leading edge of the a-wave.
